# Development of a highly sensitive luciferase assay for intracellular evaluation of coronavirus Mpro activity

**DOI:** 10.3389/fmicb.2025.1560251

**Published:** 2025-04-02

**Authors:** Bao Dong, Yuehong Chen, Xin Wang, Jing Li, Sen Zhang, Xiaoping Kang, Yuchang Li, Biao Li, Liangning Liao, Zhengwei Zhang, Jiaqi Xiong, Lele Shao, Shenghai Huang, Ye Feng, Tao Jiang

**Affiliations:** ^1^School of Basic Medical Sciences, Anhui Medical University, Hefei, China; ^2^State Key Laboratory of Pathogen and Biosecurity, Academy of Military Medical Sciences, Beijing, China; ^3^Laboratory of Advanced Biotechnology, Beijing Institute of Biotechnology, Beijing, China; ^4^School of Public Health, Mudanjiang Medical University, Mudanjiang, China; ^5^College of Veterinary Medicine, Yangzhou University, Yangzhou, China

**Keywords:** coronavirus, main protease, protease activity, reporter system, NanoBit, protease inhibitor

## Abstract

COVID-19, caused by SARS-CoV-2 virus, has emerged as a global threat to human health. The main protease (Mpro) of SARS-CoV-2 is an excellent target for the development of antiviral drugs against COVID-19, and various protease biosensors have been developed to evaluate anti-coronavirus drugs. However, the application of these protease biosensors was limited due to high background fluorescence, poor signal-to-noise ratios, and constraints in enzyme activity thresholds for accessing live viruses. In this study, we rationally designed a highly conserved Mpro cleavage site sequence among different coronaviruses (CoVs) with high proteolytic activity, and described an intracellular coronavirus Mpro proteolytic (ICMP) reporter system that takes advantage of virus-encoded Mpro expressed in infected cells to reform the NanoBiT fluorescent protein. The system can be used to visualize and identify cells infected with coronavirus, and demonstrated high compatibility with various Mpro proteins from 13 different mammalian coronaviruses (covering α, β, γ, and δ CoVs), exhibiting at least a 1,030-fold increase in luminescence. Stronger Nluc signals were detectable with CoV 229E virus infection at a MOI of 0.001. Additionally, the system proved suitable for evaluating and screening of antiviral compounds, including lufotrelvir, GC376, Nirmatrelvir, X77, MG-101, and the potential inhibitor Cynaroside. The ICMP system is not only an invaluable tool for the detection of live coronaviruses, but also for the discovery of antivirals against current and future pandemic coronaviruses.

## 1 Introduction

Coronaviruses are enveloped, single-stranded, positive-sense RNA viruses belonging to the family coronaviridae, with genomes ranging in size from 26 to 32 kilobases (Su et al., 2016; Yang and Rao, 2021). There are 86 species of coronaviruses across four genera: α, β, γ, and δ. Human coronaviruses (HCoV) include human common coronaviruses (HCoV-229E, HCoV-OC43, HCoV-NL63 and HCoV-HKU1) and high pathogenic coronaviruses such as Severe Acute Respiratory Syndrome Coronavirus (SARS-CoV), Middle East Respiratory Syndrome Coronavirus (MERS-CoV) and SARS-CoV-2 which emerged in humans in 2002, 2012, and 2019, respectively. The pandemic caused by SARS-CoV-2 had resulted in more than 776 million confirmed infections with over 7.07 million deaths (World Health Organization, 2019). The ongoing emergence of new highly pathogenic coronaviruses increases the risk of a potential future pandemic.

Targeted inhibitor development, repurposing of existing drugs, and vaccine development are crucial strategies for addressing COVID-19 and future coronavirus outbreaks (Ma et al., 2020; Baig et al., 2021; Boras et al., 2021). Currently, an important tool for high-throughput screening of antiviral drugs is the protease biosensor.

Coronaviruses encode two critical viral proteases, the main protease (Mpro) and the papain-like protease (PLpro), which are vital for the proteolytic processing of viral polyproteins. The cleavage recognition sequences are highly conserved and specific among coronaviruses, making these proteases primary targets for antiviral drug development (Zhong et al., 2009; Chen et al., 2020; Dai et al., 2020; Emrani et al., 2021). To date, several protease biosensors have been developed using coupling proteases that recognize and cleave peptide sequences for signal readout, which enable rapid screening of antiviral protease drugs. These systems do not require modifications to the virus and are compatible with various variants, as well as different types of coronaviruses. Consequently, these protease biosensors facilitate the screening of coronavirus inhibitory drugs without the need for biosafety level 3 facilities, thereby significantly advancing drug evaluation research. Current coronavirus reporting systems based on protease cleavage primarily include FlipGFP (Froggatt et al., 2020; Ma et al., 2022), Fluorescence Resonance Energy Transfer (FRET) (Brown et al., 2020; Alhadrami et al., 2021), Bioluminescence Resonance Energy Transfer (BRET) (Hou et al., 2022), and Bimolecular Fluorescent Complementation (BiFC) (Chen et al., 2022; Gerber et al., 2022). However, these coronavirus reporting systems still face challenges including high background fluorescence and low signal-to-noise ratios. Additionally, the limitations of enzyme activity thresholds in protease cleavage-dependent expression systems hinder their effectiveness in evaluating live viruses. In this study, we rationally designed a highly conserved Mpro cleavage site sequence among different coronaviruses (CoVs) with high proteolytic activity, and developed a novel cell-based protease reporter system utilizing NanoBiT for analyzing coronavirus Mpro activities and evaluation Mpro inhibitors against coronavirus infections.

## 2 Materials and methods

### 2.1 Cell and virus

HEK-293T (ATCC CRL-3216) and Huh-7 cells (JCRB0403) were maintained in Dulbecco’s modified Eagle’s medium (Gibco, New York, United States) containing 10% fetal bovine serum (FBS, Gibco), 1% penicillin–streptomycin, 2.5% HEPES and 1% non-essential amino acids at 37°C with 5% CO_2_. The coronavirus strain used in this study was HCoV-229E (Genbank accession no. PQ699976), which was amplified by culturing Huh-7 cells at 34°C in a 5% CO_2_ environment, and its viral titer was measured by the plaque assay.

### 2.2 Selection of cleavage sites

The Mpro natural cleavage site of eight coronavirus species (SARS-CoV-2, SARS-CoV, MERS-CoV, HCoV-229E, HCoV-NL63, HCoV-OC43, HCoV-HKU1, and PEDV) using Weblogo online software (Crooks et al., 2004) were conducted for a comparative analysis. A conserved peptide, LQSGF, was chosen based on previous studies, and basic amino acids residues (such as K, R) were added before this sequence to create distinct peptide fragments to serve as cleavage sites. These sequences (F1–F20) were synthesized using different primers (see Supplementary Table 2) with Site-directed mutagenesis method and then incorporated into the FlipNluc (Arakawa et al., 2023) report system, followed by cloning into the pcDNA3.1(+) vector.

HEK-293T cells were seed in polylysine-treate 96-well plates and cultured overnight. Then 20 FlipNluc reporter plasmids, along with the pcDNA3.1(+) vector expressing the SARS-CoV-2 Mpro, were co-transfected into HEK-293T cells, with no SARS-CoV-2 Mpro as negative control. The luciferase activity was measured by Nano-Glo luciferase assay system (N1120, Promage) 24 h after transfection. The highest luminescence ratio between test wells and the negative control wells was identified to initially screen for the Mpro cleavage sequence. Then, the screened plasmids were co-transfected with the pcDNA3.1(+) vectors expressing the Mpro proteins of several coronaviruses in HEK-293T cells to evaluate the strongest broad-spectrum activity of the consensus sequences for Mpro cleavage.

### 2.3 Construction and functional validation of coronavirus Mpro reporter system

The Mpro cleavage sequence F10 (VAKLQSGF) was enployed to develop a novel intracellular coronavirus Mpro proteolytic (ICMP) reporter system, which was adapted from a cell-based caspase protease reporter system (Li et al., 2022). A catalytic inactivation mutant (C145A) of C-terminus on Mpro genes (Muramatsu et al., 2016; Zhang et al., 2020), designed as a negative control, was generated by site-directed mutagenesis using the Mut Express II Fast Mutagenesis Kit V2 with the pcDNA3.1(+)-SARS-CoV-2-nsp5 plasmid as a template. All gene fragments were optimized for human codons and commercially synthesized (Sangon, China). Additionally, a similar protease reporter system with the tobacco etch virus (TEV) protease cleavage sequence (Dougherty et al., 1989) served as the mock control.

HEK-293T cells were seeded in Polylysine-treated 96-well plates at 2.5 × 10^^4^ cells/well and cultured overnight. Upon reaching 70% confluence, cells were transfected using Lipofectamine 3,000 transfection reagent (L3000015, ThermoFisher). A 1:1 mixture of 50 ng Mpro plasmid and 50 ng ICMP reporter plasmid was combined with the liposome system for 15 min and applied to each well. Negative and irrelevant controls were also transfected. Luciferase activity was measured at 24 and 48 h post-transfection.

HEK-293T cells were lysed in a solution containing cell lysate (RIPA) and phenyl methyl sulfonyl fluoride (PMSF) for western blot analysis 24 h post-transfection. Proteins were mixed with loading buffer, denatured in boiling water for 15 min, electrophoresed in a 15% Tris-Glycine PAGE Gel, and transferred to a PVDF membrane. The membrane was blocked with 5% non-fat dried milk for 1 h, incubated with primary antibodies (anti-HA antibody (100028-MM10, Sino Biological), Anti-LgBiT Monoclonal Antibody (N7100, Promega), anti-β-actin (TA-09, ZSGB-BIO)) overnight at 4°C, washed with TBST, and then incubated with a conjugated secondary antibody (ZB-2305, ZSGB-BIO). Immunoreactive proteins were detected using SuperSignal West Pico PLUS Chemiluminescent Substrate (34,577, ThermoFisher).

Furthermore, 50 ng ICMP reporter plasmid and varying dilutions (1:1–1:32) of SARS-CoV-2 Mpro were co-transfected into HEK-293T cells for 24 h to determine whether ICMP luciferase activity was dose-dependent on Mpro expression.

### 2.4 Validation of ICMP for compatibility with different coronavirus

Due to the high conservation of the amino acid sequence in the main protease (Mpro) of coronaviruses, human codon-optimized fragments of 13 coronavirus Mpro genes (Supplementary Table 3) were cloned into the pcDNA3.1(+) vector. A C145A mutation at the C-terminus of Mpro was included as a negative control. Then HEK-293T cells were seeded in 96-well plates that had been treated with Polylysine and were cultured overnight. 50 ng of an ICMP plasmid were co-transfected into 293T cells at a 1:1 ratio with 13 coronavirus Mpro expression plasmids separately. Luciferase activity was measured at 24 and 48 h post-transfection.

### 2.5 Cell viability assay

Cell viability was measured using the CellTiter-Glo Luminescent Cell Viability Assay (Promega) according to the manufacturer’s instructions. HEK-293T cells were seeded in 96-well plates, and the drug-containing DMEM medium was changed after the cells were cultured for 24 h. After 24 h, the 96-well plates were equilibrated with CellTiter-Glo reagent for 30 min at room temperature, 100 μl of the reagent was added to each well, and after mixing for 2 min using a fixed-track oscillator and incubating at room temperature for 10 min, 100 μl was taken and transferred to the white 96-well plate, and the luciferase activity was measured using the Nano-Glo Luciferase Assay System, with the time set at 1 s.

### 2.6 Intracellular detection of coronavirus 229E Mpro activity

Huh-7 cells were seeded in 96-well plates at a density of 2 × 10^4^ cells/well the day before transfection. Then cells were transfected with 100 ng of ICMP plasmid per well using Lipofectamine 3,000 transfection reagent. The ICMP plasmid with TEV protease cleavage sequence was employed as the control. The cells were infected with human coronavirus (HCoV-229E) at varying multiplicities of infection (MOIs) after 24 h post-transfection, along with uninfected cells as the negative control. Luciferase activity in half of the cells in the plate was measured at 48 and 72 h post-infection using the Nano-Glo Luciferase Assay System. These cells were first fixed by incubation in 4% paraformaldehyde (PFA) for 20 min. Then the cells were permeabilized using Perm/Wash buffer (BD Pharmingen, catalog number 554723) for 15 min and then washed with phosphate-buffered saline (PBS). The cells were subsequently incubated with a primary antibody specific to the spike S1 protein of human coronavirus (HCoV-229E), rabbit polyclonal antibody (Sino Biological, catalog number 40601-T62) overnight at 4°C. After washing, the cells were stained with Alexa Fluor 488-labeled goat anti-rabbit IgG secondary antibody (Abcam, catalog number ab15007) for 1 h. Finally, the cells were mounted with 100 μl/well of Fluoroshield with DAPI (F6057, Sigma), and fluorescence was analyzed using a Gen5 instrument (BioTek, United States).

To determine the relationship between HCoV-229E infection and luciferase activity, luminescence signals were detected at 48 h post-infection. Concurrently, total RNA was extracted from infected Huh-7 cells using the PureLink RNA Mini Kit (12183018A, ThermoFisher). The extracted RNA was then subjected to one-step real-time RT-PCR using the One Step PrimeScript RT-PCR Kit (RR064A, TaKaRa) in CFX Opus 96 Real-Time PCR System (Bio-Rad), with the following primers: forward primer 5′-CAGTCAAATGGGGCTGATGCA-3′, reverse primer 5′-AAAGGGCTATAAAGAGAGAATAAGGTATTCT-3′, and probe FAM-CCCTGACGACCACGTTGTGGTTCA-BHQ1. Total RNA as a template, qRT-PCR was performed using the One Step TB Green® PrimeScript™ PLUS RT PCR Kit (RR096A, TaKaRa) on the CFX Opus 96 Real-Time PCR System (Bio-Rad) using Mpro-F and Mpro-R primers specific for the Mpro gene of HCoV 229E. Mpro-F: ATGATGGTTGTGCTCAGGGT and Mpro-R: TTGTAGCCAGGAGAACCACAC primers specific for the Mpro gene. GAPDH was amplified as an internal control to normalize the data.

### 2.7 Anti-coronavirus infection activity of candidate Mpro inhibitors

Huh-7 cells were seeded in 96-well plates 1 day before transfection and then transfected with 100 ng of ICMP plasmid as previously described. After 24 h post-transfection, the cells were infected with HCoV-229E at an MOI of 0.1, along with uninfected cells as the control. The viral medium was replaced with culture medium containing varying concentrations (0, 0.01, 0.1, 1, 10, 100 μM) of candidate Mpro inhibitors (lufotrelvir, GC376, nirmatrelvir, X77, MG-101, Boceprevir and Cynaroside) and varying concentrations (0, 0.01, 0.1, 6.25, 12.5, 25, 50, 100 μM) of potential inhibitor cymaroside an hour post-infection. The luciferase activity and viral RNA were measured 48 h post-infection. The half effective inhibitory concentration (IC_50_) of the drug was assessed by fitting a nonlinear regression curve for the inhibition rate of the drug on luciferase expression, which was calculated as: Inhibition % = 100% × (mean of positive controls − sample value) ÷ (mean of positive controls − mean of negative controls). A lower IC50 value indicated a more potent antiviral effect of the candidate Mpro inhibitors.

### 2.8 Statistical analysis

Data was presented as the means ± SDs from at least three independent experiments. General data manipulation was conducted using Microsoft Excel, and statistical analysis using GraphPad Prism software version 9.0 (GraphPad Software, San Diego, USA). Unless otherwise stated, sample means were compared by one-way ANOVA followed by Tukey’s multiple comparison test. To compare the luminescent signals with the results of automated microscopy and 229E viral RNA expression levels, linear regression and Pearson’s correlation coefficient were used. To calculate half-maximal IC_50_, inhibition was analyzed using the [inhibitor] vs. response-Variable slope (four parameters) function.

## 3 Results

### 3.1 Screening and validation of Mpro cleavage sites

**Figure 1:**
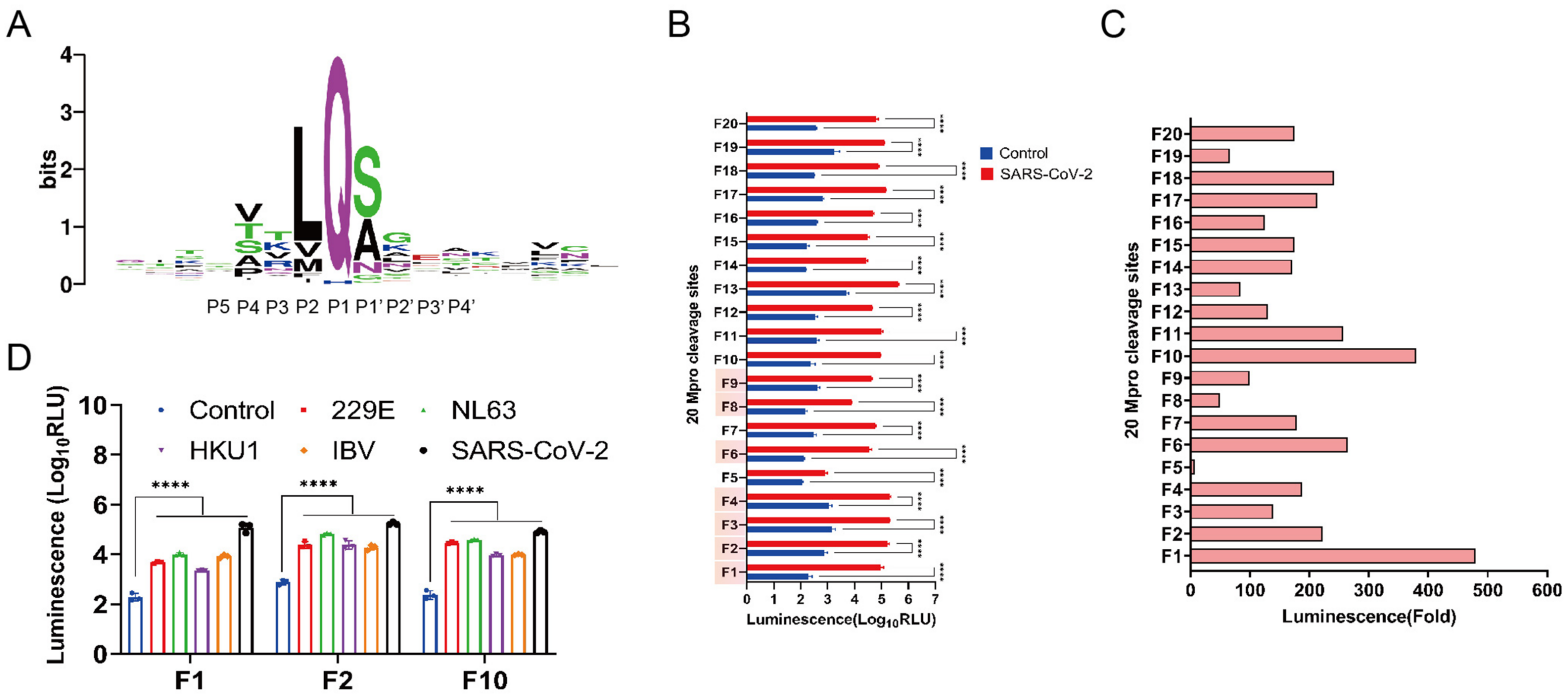
Screening for highly conserved pan-coronavirus Mpro cleavage sequences. **(A)** Sequence logo of the conservation of amino acids from multiple alignments of 88 main protease (Mpro) cleavage site sequences (weblogo.org). The relative height of the letters represents their frequency in the consensus. The total height of a logo position corresponds to the degree of conservation in the corresponding multiple-sequence alignment. The 10 residues of P5 to P4’ positions represented the Mpro recognition site. **(B)** Characterization of Nluc activity of FlipNluc reporter systems co-transfected with SARS-CoV2 Mpro *in vitro* (*n* = 3). Data were analyzed using two-way ANOVA and error bars represent the mean ± standard error (****, *p* < 0.0001). The cleavage sites (F1-F4, F6, and F7-F8 with red background on the Y-axis) which were reported in the previous studies were included in the study for analysis. **(C)** Signal-to-noise ratio (SNR) of 20 FlipNluc reporter systems. **(D)** Characterization of Nluc activity of FlipNluc reporter systems (F1, F2, and F10) with the best SNR co-transfected with 229E, NL63, HKU1, IBV or SARS-CoV2 Mpro, separately (*n* = 3). Data were analyzed using two-way ANOVA and error bars represent the mean ± standard error (****, *p* < 0.0001).

To acquire Mpro cleavage sites that are highly conserved in the coronaviridae family, we analyzed the 88 natural Mpro cleavage sites from eight species of mammalian coronaviruses, as depicted in Figure 1A. The luminescence intensities of 13 designed conserved Mpro cleavage sequences, along with 7 previously published sites, were assessed within the FlipNluc reporter system in the presence of SARS-CoV-2 Mpro. The results indicated that sites F1 (VARLQ↓SGF) and F10 (VAKLQ↓SGF) exhibited the highest cleavage activity and the greatest signal-to-noise ratio (Figures 1B,C). As shown in Figure 1D, two main Mpro cleavage sites were selected for comprehensive comparative analysis, with the structurally similar F2 (VAVLQ↓SGF) included as a reference. Notably, the F10 cleavage site, identified through systematic screening, demonstrated unique dual compatibility: it not only maintained optimal activity with SARS-CoV-2 Mpro but also exhibited broad compatibility with other coronaviruses. Functional evaluation revealed that although F1 generated slightly stronger activation signals in SARS-CoV-2 Mpro experiments, F10 showed superior broad-spectrum applicability across multiple coronaviruses. Despite F2’s similar broad-spectrum potential, its significantly reduced activation efficiency in the SARS-CoV-2 system rendered it unsuitable as a preferred candidate for pan-coronavirus applications. These comprehensive findings positioned F10 as a novel and balanced Mpro cleavage site, achieving an optimal balance between sensitivity and broad-spectrum compatibility.

To elucidate the molecular mechanism underlying the altered catalytic activity at the cleavage site, we conducted molecular dynamics (MD) simulations of SARS-CoV-2 main protease (Mpro) complexes with various substrate peptides (n = 17). Energetic analysis revealed that the F10 substrate peptide formed the most thermodynamically stable complex with SARS-CoV-2 Mpro, demonstrating the lowest potential energy and favorable conformational stability among all tested peptides (Supplementary Figure 2b). Furthermore, comparative analysis of seven coronavirus Mpro complexes with the conserved substrate peptide VAKLQSGF demonstrated hierarchical stability patterns: SARS-CoV-2 Mpro exhibited superior complex stability followed by avian infectious bronchitis virus, with human coronavirus HKU1 showing the least stable interactions (Supplementary Figure 2c).

### 3.2 Construction and functional validation of the ICMP reporter system

**Figure 2:**
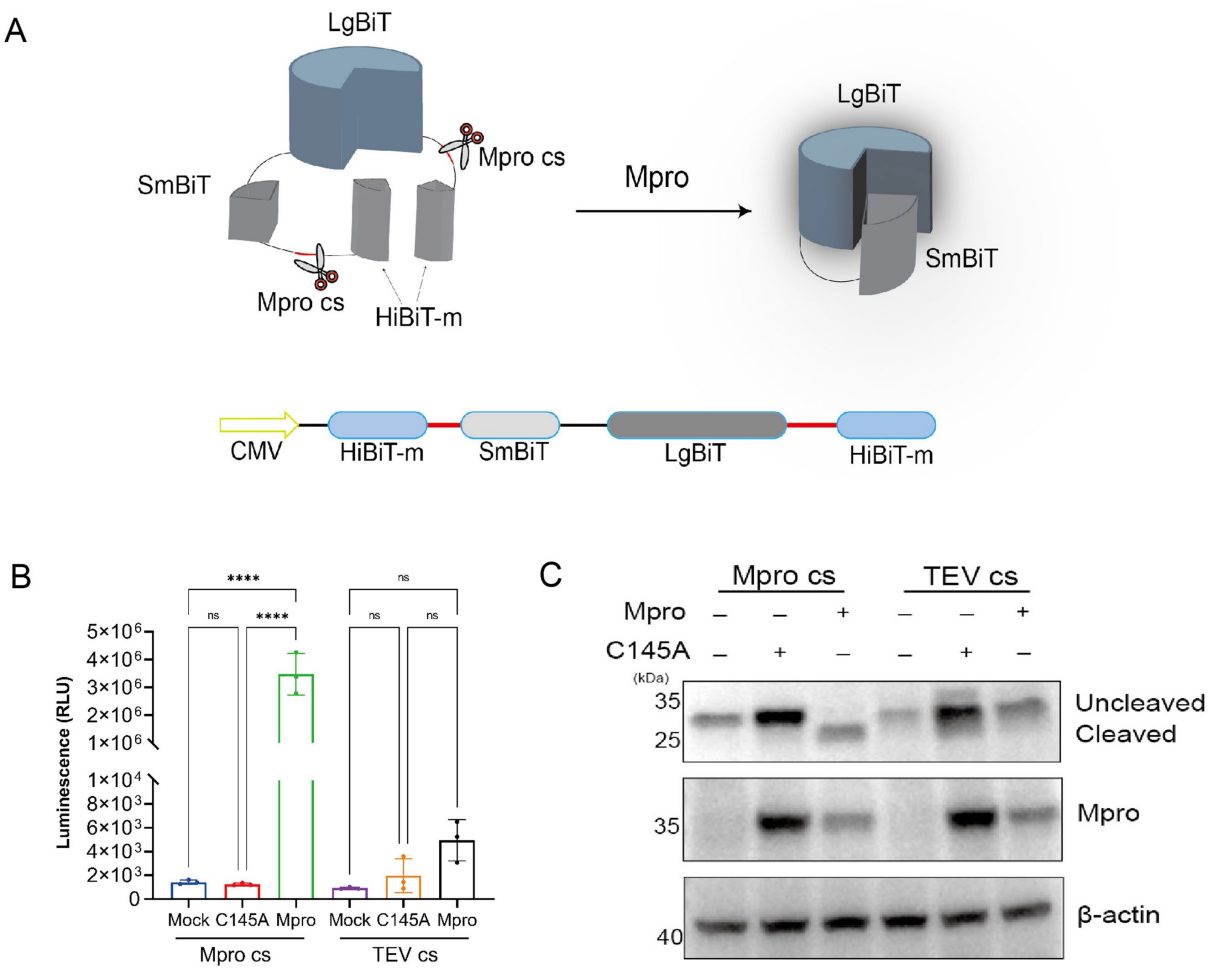
Development and characterization of the ICMP reporter system to evaluate the cleavage activity of pan-coronavirus Mpro. **(A)** Schematic representation of the ICMP reporter gene with coronavirus Mpro cleavage site (Mpro cs) based on a NanoBIT system (Promega). The small Bit (SmBiT) subunit is linked to the large Bit (LgBiT) subunit tagged with HiBiT-m to both the N and C terminus through the Mpro cleavage site. The Mpro cuts off the HiBit-m and leads to the combination of SmBiT and LgBiT, forming the functional NanoBiT® enzyme that produces bright luminescence. **(B)** HEK-293T cells were co-transfected with the ICMP reporter plasmid Mpro. The luciferase activity was measured 24 h after transfection. A significant increase in fluorescent signal was detectable only in the cells expressing catalytically active Mpro and the corresponding cleavage site. **(C)** Western blotting of co-transfected cells lysates for Mpro and NanoBiT 24 h after transfection. Cleaved smaller band of SmBiT-LgBiT was detectable only in the cells expressing the catalytically active Mpro and the corresponding cleavage sites. β-actin was used as internal control.

The SmBiT fragment was successfully ligated to the LgBiT fragment, with the first and last ends of each fragment joining a segment of the HiBiT mutant via the coronavirus Mpro cleavage site. This design preserved the original high affinity of the HiBiT mutant, but the fusion with LgBiT did not confer LgBiT’s enzymatic activity. Consequently, in the presence of the appropriate Mpro enzyme, the linker containing the cleavage site is cleaved, enabling the LgBiT fusion with SmBiT to regain enzymatic activity. We have termed this system the intracellular coronavirus Mpro proteolytic system (ICMP), as depicted in Figure 2A. The ICMP plasmid was co-transfected with the expression plasmid encodes SARS-CoV-2 Mpro. The luciferase activity increased by 3,000-fold compared to the Mock group 24 h after transfection. Additionally, we introduced a C145A mutant as a negative control, which led to no discernible increase in luminescence signal (Figure 2B). The ICMP plasmid with TEV protease cleavage sequence (ENLYFQ↓S) was employed as the control, which showed no significant change in luciferase activity when co-transfected with either Mpro or C145A Mpro mutant plasmid (Figure 2B). Western blotting results of co-expression of ICMP and Mpro plasmid indicated that the cleaved protein (23 kD) rather than the full-length protein (28 kD) of the ICMP reporter gene was detected. While neither the ICMP plasmid co-expressed with the Mpro C145A mutant nor the control with TEV protease cleavage sequence co-expressed with the SARS-CoV-2 Mpro or Mpro C145A mutant plasmid were cleaved (Figure 2C). These results demonstrated that ICMP can be effectively and specifically cleaved by Mpro.

### 3.3 Conservation validation of the ICMP reporter system based on Mpro cleavage sites

**Figure 3:**
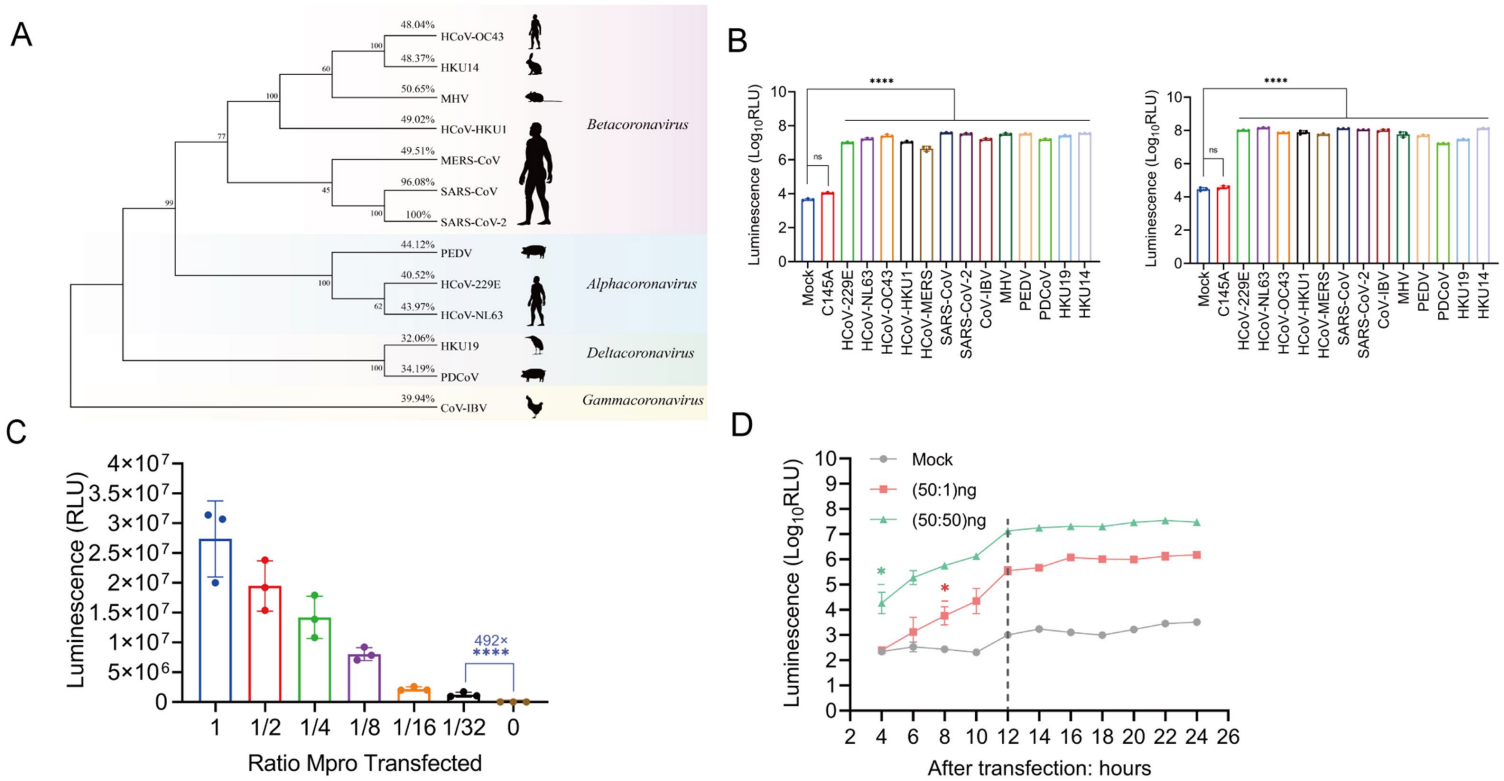
The ICMP reporter system is compatible with nearly most species of coronaviruses. **(A)** Phylogenetic tree of Mpro protein from 13 coronaviruses (α, β, γ, δ). Bootstrap values and percentage of amino acid similarity are listed at each node and show cartoons of the main host species on the right side. **(B)** Characterization of luminescence signals in 293T cells co-transfected with ICMP reporter plasmid and different coronavirus Mpro 24 h (left) and 48 h (right) after transfection. **(C)** Characterization of luminescence signals in 293T cells transfected with different amounts of SARS-CoV-2 Mpro 24 h after transfection. The luminescence signal was decreased, agreeably to the amount of transfected Mpro plasmid DNA. **(D)** The ICMP reporter gene was co-transfected into cells with different amounts of SARS-CoV-2 Mpro plasmid, and luciferase signals were measured every 2 h from 4 to 24 h post-transfection. The 1:1 ratio of ICMP to Mpro plasmid DNA differed 4 h after transfection, while the 50:1 ratio of ICMP to Mpro DNA differed 8 h after transfection. The grey dotted line indicates the luminescence values of mock group. Data were analyzed using one-way ANOVA and error bars represent the mean ± standard error (*n* = 3; *, *p* < 0.05, ****, *p* < 0.0001).

Phylogenetic and identity analysis revealed that Mpro is fairly conserved across the Coronaviridae family, as depicted in Figure 3A. The ICMP reporter system was co-transfected with 13 different coronavirus Mpro constructs. All coronavirus Mpro were observed to elicit luminescence from the reporter system when compared to the mock control 24 h post-transfection. The luminescence signal intensities demonstrated a significant increase, ranging from 1,030-fold to 8,330-fold, as illustrated in Figure 3B (left panel). At 48 h post-transfection, the signal intensity exhibited a substantial increase varying from 929 to 4,700-fold, compared to the previous study which reported about only 50-fold increase, as shown in Figure 3B (right panel). No significant increase in the luminescence signal was detected in the negative control.

Furthermore, the luciferase activity demonstrated a positive correlation with the quantity of Mpro transfections, indicating that ICMP luciferase activity was dose-dependent on Mpro expression (Figure 3C). Notably, even when the concentration of SARS-CoV-2 Mpro protein was reduced to 1.5625 ng (1:32 dilution), the luciferase activity of 10^5^ relative luminescence units (RLU) represented an impressive enhancement of 492-fold in comparison to the negative control group. This result underscored the high sensitivity and responsiveness of the ICMP system to Mpro (Figure 3C). Next, we transfected the ICMP and SARS-CoV-2 Mpro at different transfection ratios and monitored the luciferase activity at 2-h intervals starting at 4 h after transfection. During the 20 h transfection time course, the luciferase signals increased significantly from 4 h post-transfection at a ratio of 1:1 and from 8 h post-transfection at a ratio of 50:1 (Figure 3D). The results indicated that the ICMP reporter system was able to constantly detect the change in the luciferase signal and even when Mpro was diluted by 50-fold.

### 3.4 Evaluation of ICMP-based assay for quantification of coronavirus infection

**Figure 4:**
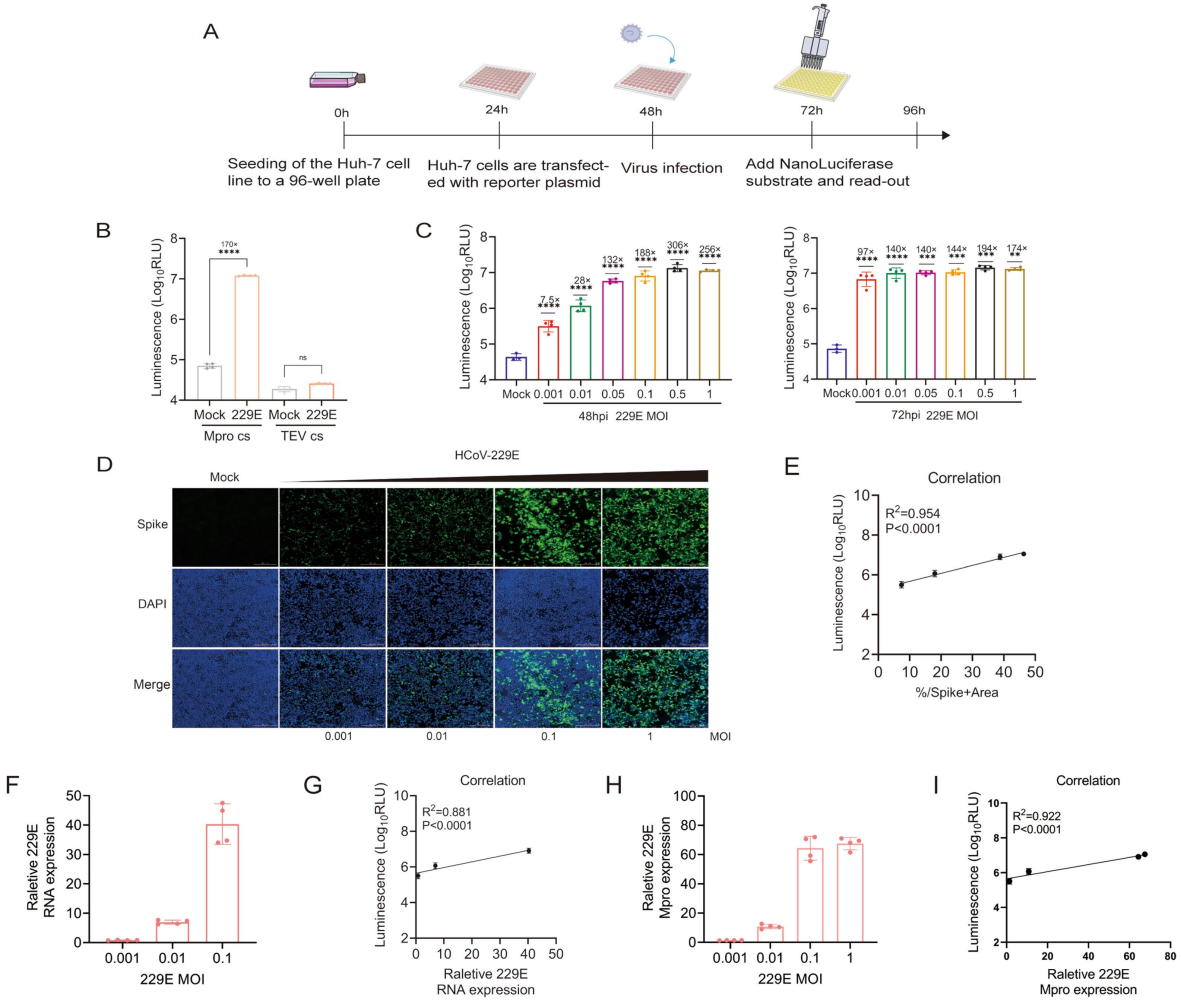
ICMP reporter system to monitor coronavirus infections. **(A)** Schematic representation of quantification of coronavirus infection with the luminescence signal. **(B)** HCoV 229E viruses were used to infect Huh-7 cells which were transfected with ICMP plamsid or the control reporter plasmid containing the TEV cleavage site (M.O.I = 0.1), and luminescence signals were measured 48 h post-infection. The uninfected cells with HCoV 229E were used as control (mock) group. **(C)** ICMP transfected Huh7 Cells were infected with HCoV 229E virus at different MOI and luminescence signals were measured at 48 and 72 h post-infection. **(D)** Immunofluorescence staining for Spike protein (green) in DAPI-stained (blue) nuclei of ICMP-transfected Huh7 cells infected with HCoV 229E virus 48 h post-infection. **(E)** Correlation between the Nluc luminescence and the proportions of HCoV 229E spike protein determined by IFA during virus infection, was analyzed by Pearson (R^2^). **(F)** Quantification of viral RNA in HCoV 229E-infected cell lysates 48 h after infection. Data shows RNA levels relative to GAPDH. **(G)** Correlation between the Nluc luminescence and viral RNA dynamics during HCoV 229E infection was analyzed by Pearson (R^2^). **(H)** Quantification of viral Mpro in HCoV 229E-infected cell lysates 48 h after infection. Data shows RNA levels relative to GAPDH. **(I)** Correlation between the Nluc luminescence and viral Mpro dynamics during HCoV 229E infection was analyzed by Pearson (R^2^). Data were analyzed using one-way ANOVA and error bars represent the mean ± standard error (*n* = 4; **, *p* < 0.01, ***, *p* < 0.001, ****, *p* < 0.0001, ns, *p* > 0.05).

The luminescence activity exhibited a significant increase in infected cells that were transfected with the ICMP reporter plasmid, whereas low luminescence signals were detected in the non-cleavable control (Figures 4A,B). To further investigate whether the ICMP system could be activated by a low dose of HCoV-229E virus infection, different multiplicities of infection (M.O.Is) were tested. Compared to uninfected cells, infected cells exhibited a significant increase in luminescence at 24 h post infection, 5-fold increase in luminescence signal at MOI of 0.5 (Supplementary Figure 4). It was worth noting that luminescence was detectable at the lowest MOI of 0.001 at 48 h post-infection. A greater dynamic range was observed at 72 h post-infection, with at least 97-fold increase in luminescence compared to uninfected cells (Figure 4C). The intensity of the luminescent signal demonstrated a positive correlation with the quantity of virus. Additionally, the results of the immunofluorescence assay indicated a strong correlation between the luminescent signal and the proportion of infected cells that were positive for the spike protein (Figures 4D,E). The RT-qPCR results indicated that as the concentration of inoculated 229E virus increases, there was a corresponding increase in viral RNA levels within the infected Huh-7 cells (Figure 4F). The expression of luminescence and the quantity of viral RNA in 229E-infected cells exhibited a significant positive correlation (R^2^ = 0.881, *p* < 0.0001), normalized to GAPDH 48 h post-infection (Figure 4G). Concurrently, the intracellular expression levels of the 229E Mpro were assessed. The data indicated that Mpro expression escalated in tandem with the viral load. Moreover, there was a positive correlation (R^2^ = 0.922, p < 0.0001) between luciferase activity and Mpro expression (Figures 4H,I). These results indicated that the ICMP-based assay can effectively quantify coronavirus infection.

### 3.5 Evaluation of antiviral activity of Mpro inhibitors by ICMP-based assay

**Figure 5:**
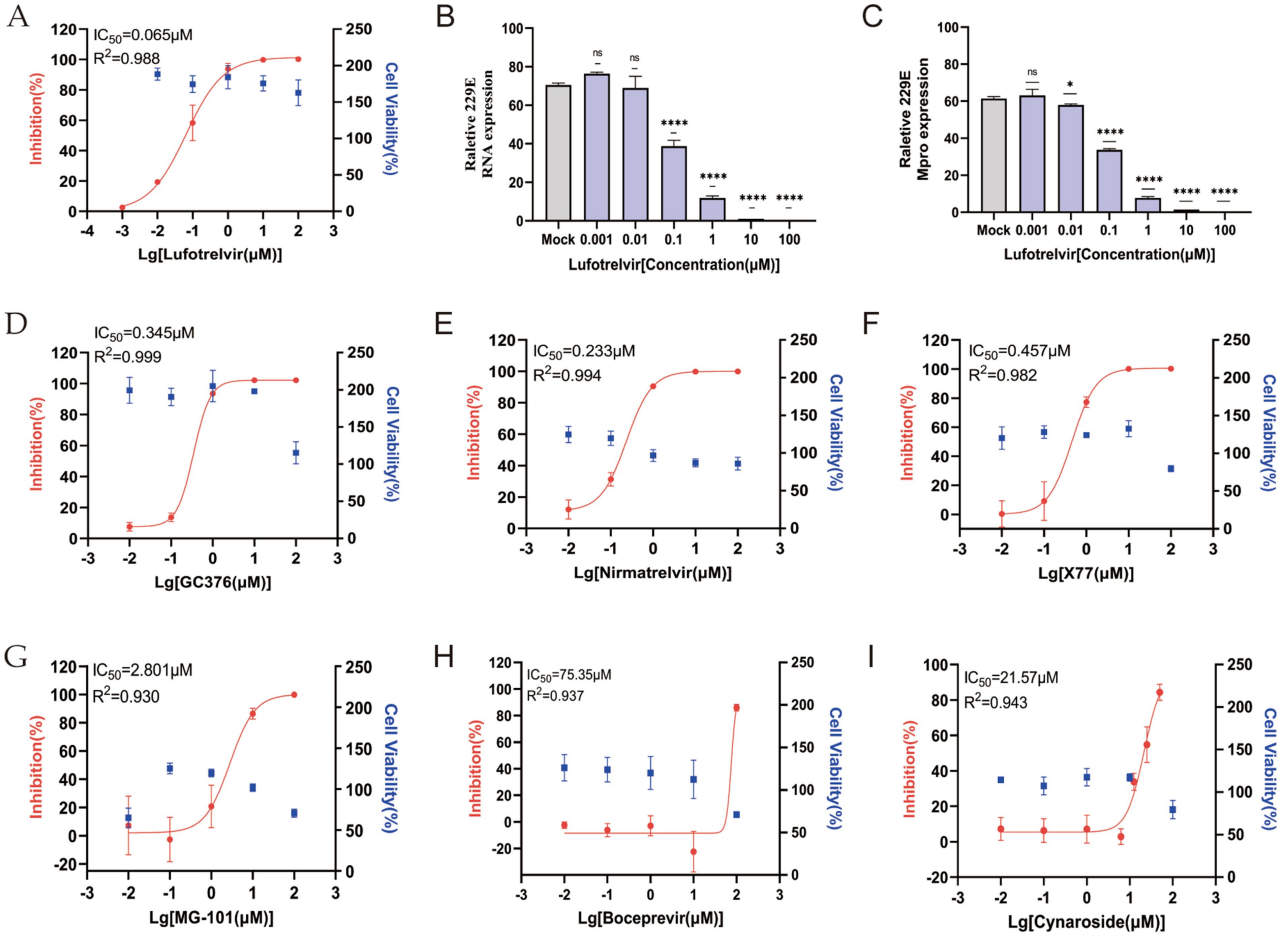
Evaluation of in vitro antiviral activity of Mpro inhibitors against HCoV 229E virus in the ICMP reporter system-based assay. **(A)** Dose–response curves and IC_50_ for Lufotrelvir against 229E virus infection (M.O.I = 0.1). **(B)** Relative HCoV 229E viral RNA levels, normalized to GAPDH, in the infected cells treated with Lufotrelvir. Data are shown as means ± SD. *p* values were analyzed by one-way ANOVA (*n* = 4, ****, *p* < 0.0001; ns, *p* > 0.05). **(C)** Relative HCoV 229E viral Mpro levels, normalized to GAPDH, in the infected cells treated with Lufotrelvir. Data are shown as means ± SD. *p* values were analyzed by one-way ANOVA (n = 3, *, *p* < 0.05; ****, *p* < 0.0001; ns, *p* > 0.05). **(D–I)** Dose–response curves for GC376, Nirmatrelvir, X77, MG-101, Boceprevir and Cynaroside are shown in red. Cell viability (blue) were accessed relative to vehicle-only (DMSO) samples. IC_50_ and R-squared values were estimated by fitting a nonlinear regression curve to the data from each individual experiment for each inhibitor.

The impact of various candidate Mpro inhibitors on HCoV-229E infection was assessed in cells transfected with ICMP plasmid. Lufotrelvir (PF-07304814), a broad-spectrum coronavirus 3CL protease inhibitor, demonstrated potent antiviral activity with an IC_50_ value of 0.065 μM at 48 h post-infection (Figure 5A). This was observed alongside a negative correlation bet ween viral RNA levels, as detected by RT-qPCR, and increasing drug concentrations (Figure 5B), aligning with previous studies (Boras et al., 2021). And intracellular 229E Mpro expression decreased significantly with the increasing drug concentration (Figure 5C). Subsequently, four inhibitors, GC376, nirmatrelvir, X77, and MG-101 (Vangeel et al., 2022; Zhang et al., 2024), known for their inhibitory effects on SARS-CoV-2 Mpro were evaluated. Their respective IC_50_ values were 0.345, 0.233, 0.457, and 2.801 μM, as depicted in Figures 5D–G. The result underscore the differential efficacy of these protease inhibitors against HCoV-229E. Boceprevir, known for its broad-spectrum antiviral activity against SARS-CoV-2 and other human coronaviruses, was also tested by using the ICMP assay. However, it showed limited antiviral potency with IC_50_ values exceeding 50 μM (Figure 5H), consistent with previous studies (Franko et al., 2021; Hu et al., 2021). Then, cynaroside, a potential inhibitor of SARS-CoV-2 Mpro, was examined for its inhibitory effect on 229E infection. It exhibited a moderate inhibitory effect with an IC_50_ value of 21.57 μM (Figure 5I).

## 4 Discussion

The ongoing COVID-19 pandemic underscores the necessity for proactive development of tools capable of addressing potential future emergence of novel coronaviruses. For example, Froggatt et al. (2020) developed a high-throughput screening method based on FlipGFP, targeting 3CLpro cleavage sites to identify SARS-CoV-2 protease inhibitors. This method relied on the conformational flipping of isolated GFP and the reconstitution of fluorescent motifs, enabling the screening of a diverse range of SARS-CoV-2 protease inhibitors (Ma et al., 2021; Ma et al., 2022). Arakawa et al. (2023) modified the system by replacing EGFP with Nluc, developing FlipNluc for applications in apoptosis detection. However, these studies were not designed for direct viral infection detection, and subsequent research demonstrated that the FlipGFP system was unsuitable for this purpose, as it either failed to detect viral infection or showed only minimal differences upon infection. Instead, these systems were proved to be highly effective for screening cleavage sites. Among various targets, Mpro and PLpro, a highly conserved protease across coronaviruses, stands out as a key focus for drug development against SARS-CoV-2. Mpro, a 306-amino acid dimeric protein, is shorter than PLpro, facilitating the screening of cleavage sites, hence the prevalence of Mpro-based protease biosensors in current antiviral drug designs. Our study designed a cell-based reporter system named ICMP which was based on the optimized Mpro cleavage sequence. In previous studies, the relative rates of cleavage of different substrate sequences correlated with different structural properties, that is a preference for small hydrophobic residues at the P4 position, a preference for positively charged residues at the P3 position, a preference for hydrophobic residues without a β-branch at P2, and Gln as the best residue at the P1 position (Chuck et al., 2011). In addition, cleavage of the peptide bond between P1 and P1’ positions were catalyzed by the Cys145 and His41 dyad (Chen et al., 2005). Guided by this principle, we designed coronavirus Mpro recognition sites and, in this study, employed the FlipNluc system to screen 13 self-designed Mpro cleavage sequences alongside 7 previously published sites. The results indicated that sites F1 and F10 exhibited the highest cleavage activity and the greatest signal-to-noise ratio. The luciferase activity assay demonstrated that the F10 cleavage site exhibits both a high signal-to-noise ratio and broad-spectrum compatibility, demonstrating superior performance compared to other cleavage sites. Furthermore, molecular dynamics (MD) simulations revealed that the F10 substrate peptide (VAKLQSGF) forms a structurally stable interaction with SARS-CoV-2 Mpro, as evidenced by its lowest binding energy. This thermodynamic stability is likely achieved through optimal electrostatic complementarity between the Glu47 residue and the P3Lys side chain. Notably, prior investigations have established that the lysine residue at the P3 position confers greater specificity than arginine, an observation attributable to the critical role of electrostatic interactions between Glu47 and P3Lys in mediating selective substrate recognition (Phakthanakanok et al., 2009). Since the F10 cleavage site demonstrated potential compatibility with all tested coronaviruses, it was selected as the optimal sequence for establishing the ICMP system. To achieve this, we integrated the optimal sequence into a cell-based reporter system, which was subsequently named ICMP.

This system involves the fusion of SmBiT and LgBiT fragments, which were originally designed for the detection of caspase activity (Li et al., 2022). Both ends of this fusion were linked to segments of the HiBiT mutant via the SARS-CoV-2 Mpro cleavage site. In the presence of the appropriate Mpro enzyme, the linker containing the cleavage site is hydrolyzed, enabling LgBiT to associate with SmBiT and thus gain enzymatic activity. Our results confirm that ICMP can effectively detect 13 different species of coronaviruses, including the SARS-CoV-2. In fact, most current reporter systems are specifically designed for detecting the SARS-CoV-2, with few being universally effective on detecting different coronaviruses. This limitation is largely due to the fact that common coronaviruses exhibit weaker replication capabilities and lower Mpro protein expression levels compared to SARS-CoV-2. Consequently, the development of systems for detecting common coronaviruses necessitates a high degree of sensitivity. The ICMP system exhibited a significant increase in luminescence when co-transfected separately with 13 different coronavirus Mpro constructs. All Mpro demonstrated at least a 1,030-fold enhancement, significantly exceeding the 100-fold enhancement reported in previous studies. Furthermore, stronger signals were detected by the ICMP system by infection of HCoV 229E virus at a low MOI of 0.001, demonstrating its exceptional sensitivity. This sensitivity suggests that the ICMP system has significant potential for detecting coronavirus infections.

To determine whether the ICMP system can accurately reflect the efficacy of drugs in inhibiting viral infections, we tested a series of coronavirus drugs. Lufotrelvir is a phosphate prodrug that is rapidly converted *in vivo* into the active moiety PF-00835231 (Boras et al., 2021). Lufotrelvir showed significant anti-229E coronavirus activity in a cell-based assay with an IC_50_ value of 0.065 μM, consistent with the results of the previous study (IC_50_ = 0.058 μM; Boras et al., 2021). In addition, Lufotrelvir demonstrated potent inhibitory activity against all tested coronavirus Mpro as previous study (Boras et al., 2021), confirming Lufotrelvir is a promising broad-spectrum anti-coronavirus agent. Furthermore, we evaluated the antiviral potency of three SARS-CoV-2 Mpro inhibitors, nirmatrelvir, X77, and MG-101 (Owen et al., 2021; Luttens et al., 2022; Narayanan et al., 2022; Zhang et al., 2024), against the HCoV 229E Mpro using ICMP. The results revealed that both nirmatrelvir and X77 exhibited higher antiviral potency against 229E Mpro compared to MG-101, which displayed moderate cellular inhibition. These findings indicated that the effectiveness of these inhibitors may extend to a broader spectrum of coronavirus variants besides SARS-CoV-2. Boceprevir, approved by FDA to treat HCV infection in 2011 (Malcolm et al., 2006), has shown potency to inhibit Mpro enzymatically recently. In our study, boceprevir displayed very weak inhibitory potency against HCoV 229E, which consistent with the results of previous study (Cao et al., 2022). Our study further confirmed that novel targets for COVID-19 drug development should hit on additional key steps in the SARS-CoV-2 pathogenesis and replication pathway, rather than solely inhibiting Mpro to achieve a high antiviral effect.

GC376, initially designed to target coronaviruses like feline infectious peritonitis virus (FIPV) in cats (Pedersen et al., 2018), has been repurposed by Anivive Lifesciences Inc. for the treatment of COVID-19 patients (Fu et al., 2020; Vuong et al., 2020). This drug exhibits high potency for inhibiting the Mpro in vivo. However, its efficacy in inhibiting SARS-CoV-2 *in vitro* was a subject of debate. Our study employed the ICMP assay to demonstrate that GC376 effectively suppresses the replication of the 229E virus in vitro. This inhibitory effect was mediated through the inhibition of Mpro.

To further validate the potential of the ICMP system in discovering future Mpro inhibitors, we selected an Mpro inhibitor with inhibitory potential, Cynaroside, a luteolin 7-O-β-D-glucoside, have been reported to be a potential inhibitory against the RNA polymerase of influenza viruses (Zima et al., 2020). Cynaroside has been identified as a potential inhibitor of the main protease of SARS-CoV-2 through molecular docking studies (Johnson et al., 2022; Moezzi, 2023). In the present study, we determined that cynaroside was able to effectively be inhibited the replication of the HCoV 229E virus in huh7 cells, demonstrating moderate antiviral activity. Although the exact mechanism of inhibition is not yet clear, our investigation into this possibility is likely lead to the discovery of novel COVID-19 drugs.

Currently, most drug evaluations are restricted to plasmid transfection, which fails to accurately represent the effects of therapeutic compounds on cells. In fact, the effects of identified protease inhibitors may vary significantly when these compounds are applied to viral infections. Our ICMP system addresses this limitation by detecting coronavirus infections at very low titers and discerning the inhibitory effects of drugs through the attenuation of fluorescent signals. The ICMP system has demonstrated high effectiveness in monitoring coronavirus Mpro activity, offering a robust solution for future drug screening. While our ICMP reporter system demonstrates high sensitivity and specificity for detecting Mpro activity, several limitations should be considered. First, although we have validated the system in both 293T and Huh7 cells, showing consistent signal-to-noise ratios (thousands-fold differences) across these cell types, it remains to be determined whether the fluorescence signal might vary in other cell types or under different metabolic states. Future studies should expand the validation to include primary cells and cells with altered metabolic profiles to further assess the robustness of the system. Due to the biosafety constraints, we currently only use the HCoV 229E strain for assessment. In the future, it will be essential to expand the scope to encompass a wider variety of coronavirus strains in order to enhance our understanding and treatment of these diseases. Finally, the application of this system in more complex biological environments, such as animal models, will require careful optimization to account for potential interference from host factors. Future work should focus on addressing these limitations to enhance the translational potential of the ICMP reporter system for both basic research and clinical applications.

## 5 Conclusion

In conclusion, a cell-based coronavirus reporter system was developed to quantify the activity of Mpro or coronavirus infections. This cell-based assay is highly applicable for screening candidate antiviral compounds against both SARS-CoV-2 and any other coronaviruses. Using this assay, we identified a drug, Cynaroside, with moderate inhibitory effects against 229E virus, which may possess broad-spectrum antiviral potential. Furthermore, the assay demonstrated significant potential for applications in aiding the diagnosis and detection of emerging coronaviruses.

## Data Availability

The datasets presented in this study can be found in online repositories. The names of the repository/repositories and accession number(s) can be found in the article/Supplementary material.

## References

[ref1] Alhadrami H. A. Hassan A. M. Chinnappan R. Al-Hadrami H. Abdulaal W. H. Azhar E. I.. (2021). Peptide substrate screening for the diagnosis of SARS-CoV-2 using fluorescence resonance energy transfer (FRET) assay. Microchim. Acta 188:137. doi: 10.1007/s00604-021-04766-5, PMID: 33763734 PMC7990899

[ref2] Arakawa M. Yoshida A. Okamura S. Ebina H. Morita E. (2023). A highly sensitive NanoLuc-based protease biosensor for detecting apoptosis and SARS-CoV-2 infection. Sci. Rep. 13:1753. doi: 10.1038/s41598-023-28984-4, PMID: 36720982 PMC9887574

[ref3] Baig M. H. Sharma T. Ahmad I. Abohashrh M. Alam M. M. Dong J.-J. (2021). Is PF-00835231 a Pan-SARS-CoV-2 Mpro inhibitor? A comparative study. Molecules 26:1678. doi: 10.3390/molecules26061678, PMID: 33802860 PMC8002701

[ref4] Boras B. Jones R. M. Anson B. J. Arenson D. Aschenbrenner L. Bakowski M. A.. (2021). Preclinical characterization of an intravenous coronavirus 3CL protease inhibitor for the potential treatment of COVID19. Nat. Commun. 12:6055. doi: 10.1038/s41467-021-26239-2, PMID: 34663813 PMC8523698

[ref5] Brown A. S. Ackerley D. F. Calcott M. J. (2020). High-throughput screening for inhibitors of the SARS-CoV-2 protease using a FRET-biosensor. Molecules 25:4666. doi: 10.3390/molecules25204666, PMID: 33066278 PMC7587356

[ref6] Cao W. Cho C.-C. D. Geng Z. Z. Shaabani N. Ma X. R. Vatansever E. C.. (2022). Evaluation of SARS-CoV-2 Main protease inhibitors using a novel cell-based assay. ACS Cent. Sci. 8, 192–204. doi: 10.1021/acscentsci.1c00910, PMID: 35229034 PMC8848508

[ref7] Chen S. Chen L. L. Luo H. B. Sun T. Chen J. Ye F.. (2005). Enzymatic activity characterization of SARS coronavirus 3C-like protease by fluorescence resonance energy transfer technique. Acta Pharmacol. Sin. 26, 99–106. doi: 10.1111/j.1745-7254.2005.00010.x, PMID: 15659121 PMC7091904

[ref8] Chen K. Y. Krischuns T. Varga L. O. Harigua-Souiai E. Paisant S. Zettor A.. (2022). A highly sensitive cell-based luciferase assay for high-throughput automated screening of SARS-CoV-2 nsp5/3CLpro inhibitors. Antivir. Res. 201:105272. doi: 10.1016/j.antiviral.2022.105272, PMID: 35278581 PMC8906008

[ref9] Chen Y. Liu Q. Y. Guo D. Y. (2020). Emerging coronaviruses: genome structure, replication, and pathogenesis. J. Med. Virol. 92, 418–423. doi: 10.1002/jmv.25681, PMID: 31967327 PMC7167049

[ref10] Chuck C. P. Chow H. F. Wan D. C. Wong K. B. (2011). Profiling of substrate specificities of 3C-like proteases from group 1, 2a, 2b, and 3 coronaviruses. PLoS One 6:e27228. doi: 10.1371/journal.pone.0027228, PMID: 22073294 PMC3206940

[ref11] Crooks G. E. Hon G. Chandonia J. M. Brenner S. E. (2004). WebLogo: A sequence logo generator. Genome Res. 14, 1188–1190. doi: 10.1101/gr.849004, PMID: 15173120 PMC419797

[ref12] Dai W. Zhang B. Jiang X. M. Su H. Li J. Zhao Y.. (2020). Structure-based design of antiviral drug candidates targeting the SARS-CoV-2 main protease. Science 368, 1331–1335. doi: 10.1126/science.abb4489, PMID: 32321856 PMC7179937

[ref13] Dougherty W. G. Cary S. M. Parks T. D. (1989). Molecular genetic analysis of a plant virus polyprotein cleavage site: a model. Virology 171, 356–364. doi: 10.1016/0042-6822(89)90603-X, PMID: 2669323

[ref14] Emrani J. Ahmed M. Jeffers-Francis L. Teleha J. C. Mowa N. Newman R. H.. (2021). SARS-COV-2, infection, transmission, transcription, translation, proteins, and treatment: a review. Int. J. Biol. Macromol. 193, 1249–1273. doi: 10.1016/j.ijbiomac.2021.10.172, PMID: 34756970 PMC8552795

[ref15] Franko N. Teixeira A. P. Xue S. Charpin-El Hamri G. Fussenegger M. (2021). Design of modular autoproteolytic gene switches responsive to anti-coronavirus drug candidates. Nat. Commun. 12:6786. doi: 10.1038/s41467-021-27072-3, PMID: 34811361 PMC8609006

[ref16] Froggatt H. M. Heaton B. E. Heaton N. S. (2020). Development of a fluorescence-based, high-throughput SARS-CoV-2 3CL (pro) reporter assay. J. Virol. 94, e01265–e01220. doi: 10.1128/JVI.01265-20, PMID: 32843534 PMC7592234

[ref17] Fu L. Ye F. Feng Y. Yu F. Wang Q. Wu Y.. (2020). Both Boceprevir and GC376 efficaciously inhibit SARS-CoV-2 by targeting its main protease. Nat. Commun. 11:4417. doi: 10.1038/s41467-020-18233-x, PMID: 32887884 PMC7474075

[ref18] Gerber P. P. Duncan L. M. Greenwood E. J. D. Marelli S. Naamati A. Teixeira-Silva A.. (2022). A protease-activatable luminescent biosensor and reporter cell line for authentic SARS-CoV-2 infection. PLoS Pathog. 18:e1010265. doi: 10.1371/journal.ppat.1010265, PMID: 35143592 PMC8865646

[ref19] Hou N. K. Peng C. Zhang L. J. Zhu Y. Y. Hu Q. (2022). BRET-based self-cleaving biosensors for SARS-CoV-2 3CLpro inhibitor discovery. Microbiol. Spectr. 10:e0255921. doi: 10.1128/spectrum.02559-21, PMID: 35758897 PMC9430692

[ref20] Hu Y. Ma C. Szeto T. Hurst B. Tarbet B. Wang J. (2021). Boceprevir, Calpain inhibitors II and XII, and GC-376 have broad-Spectrum antiviral activity against coronaviruses. ACS Infect. Dis. 7, 586–597. doi: 10.1021/acsinfecdis.0c00761, PMID: 33645977 PMC7944397

[ref21] Johnson T. O. Adegboyega A. E. Ojo O. A. Yusuf A. J. Iwaloye O. Ugwah-Oguejiofor C. J.. (2022). A computational approach to elucidate the interactions of chemicals from Artemisia annua targeted toward SARS-CoV-2 Main protease inhibition for COVID-19 treatment. Front. Med. 9:907583. doi: 10.3389/fmed.2022.907583, PMID: 35783612 PMC9240657

[ref22] Li J. Wang J. L. Gan C. Y. Cai X. F. Wang Y. W. Long Q. X.. (2022). Caspase sensors based on NanoLuc. J. Biotechnol. 357, 100–107. doi: 10.1016/j.jbiotec.2022.08.005, PMID: 35963591

[ref23] Luttens A. Gullberg H. Abdurakhmanov E. Vo D. D. Akaberi D. Talibov V. O.. (2022). Ultralarge virtual screening identifies SARS-CoV-2 Main protease inhibitors with broad-Spectrum activity against coronaviruses. J. Am. Chem. Soc. 144, 2905–2920. doi: 10.1021/jacs.1c08402, PMID: 35142215 PMC8848513

[ref24] Ma C. Sacco M. D. Hurst B. Townsend J. A. Hu Y. Szeto T.. (2020). Boceprevir, GC-376, and calpain inhibitors II, XII inhibit SARS-CoV-2 viral replication by targeting the viral main protease. Cell Res. 30, 678–692. doi: 10.1038/s41422-020-0356-z, PMID: 32541865 PMC7294525

[ref25] Ma C. Sacco M. D. Xia Z. Lambrinidis G. Townsend J. A. Hu Y.. (2021). Discovery of SARS-CoV-2 papain-like protease inhibitors through a combination of high-throughput screening and a Flip GFP-based reporter assay. ACS Cent. Sci. 7, 1245–1260. doi: 10.1021/acscentsci.1c00519, PMID: 34341772 PMC8265724

[ref26] Ma C. Tan H. Choza J. Wang Y. Wang J. (2022). Validation and invalidation of SARS-CoV-2 main protease inhibitors using the Flip-GFP and protease-Glo luciferase assays. Acta Pharm. Sin. B 12, 1636–1651. doi: 10.1016/j.apsb.2021.10.026, PMID: 34745850 PMC8558150

[ref27] Malcolm B. A. Liu R. Lahser F. Agrawal S. Belanger B. Butkiewicz N.. (2006). SCH 503034, a mechanism-based inhibitor of hepatitis C virus NS3 protease, suppresses polyprotein maturation and enhances the antiviral activity of alpha interferon in replicon cells. Antimicrob. Agents Chemother. 50, 1013–1020. doi: 10.1128/AAC.50.3.1013-1020.2006, PMID: 16495264 PMC1426438

[ref28] Moezzi M. S. (2023). Comprehensive in silico screening of flavonoids against SARS-CoV-2 main protease. J. Biomol. Struct. Dyn. 41, 9448–9461. doi: 10.1080/07391102.2022.2142297, PMID: 36342071

[ref29] Muramatsu T. Takemoto C. Kim Y. T. Wang H. Nishii W. Terada T.. (2016). SARS-CoV 3CL protease cleaves its C-terminal autoprocessing site by novel subsite cooperativity. Proc. Natl. Acad. Sci. USA 113, 12997–13002. doi: 10.1073/pnas.1601327113, PMID: 27799534 PMC5135343

[ref30] Narayanan A. Narwal M. Majowicz S. A. Varricchio C. Toner S. A. Ballatore C.. (2022). Identification of SARS-CoV-2 inhibitors targeting Mpro and PLpro using in-cell-protease assay. Commun. Biol. 5:169. doi: 10.1038/s42003-022-03090-9, PMID: 35217718 PMC8881501

[ref31] Owen D. R. Allerton C. M. N. Anderson A. S. Aschenbrenner L. Avery M. Berritt S.. (2021). An oral SARS-CoV-2 M (pro) inhibitor clinical candidate for the treatment of COVID-19. Science 374, 1586–1593. doi: 10.1126/science.abl4784, PMID: 34726479

[ref32] Pedersen N. C. Kim Y. Liu H. Galasiti Kankanamalage A. C. Eckstrand C. Groutas W. C.. (2018). Efficacy of a 3C-like protease inhibitor in treating various forms of acquired feline infectious peritonitis. J. Feline Med. Surg. 20, 378–392. doi: 10.1177/1098612X17729626, PMID: 28901812 PMC5871025

[ref33] Phakthanakanok K. Ratanakhanokchai K. Kyu K. L. Sompornpisut P. Watts A. Pinitglang S. (2009). A computational analysis of SARS cysteine proteinase-octapeptide substrate interaction: implication for structure and active site binding mechanism. BMC Bioinf. 10:S48. doi: 10.1186/1471-2105-10-S1-S48, PMID: 19208150 PMC2648740

[ref34] Su S. Wong G. Shi W. Liu J. Lai A. C. K. Zhou J.. (2016). Epidemiology, genetic recombination, and pathogenesis of coronaviruses. Trends Microbiol. 24, 490–502. doi: 10.1016/j.tim.2016.03.003, PMID: 27012512 PMC7125511

[ref35] Vangeel L. Chiu W. De Jonghe S. Maes P. Slechten B. Raymenants J.. (2022). Remdesivir, Molnupiravir and Nirmatrelvir remain active against SARS-CoV-2 omicron and other variants of concern. Antivir. Res. 198:105252. doi: 10.1016/j.antiviral.2022.105252, PMID: 35085683 PMC8785409

[ref36] Vuong W. Khan M. B. Fischer C. Arutyunova E. Lamer T. Shields J.. (2020). Feline coronavirus drug inhibits the main protease of SARS-CoV-2 and blocks virus replication. Nat. Commun. 11:4282. doi: 10.1038/s41467-020-18096-2, PMID: 32855413 PMC7453019

[ref37] World Health Organization (2019). Coronavirus (COVID-19) dashboard. Available online at: https://covid19.who.int/ (Accessed December 16, 2024).

[ref38] Yang H. Rao Z. (2021). Structural biology of SARS-CoV-2 and implications for therapeutic development. Nat. Rev. Microbiol. 19, 685–700. doi: 10.1038/s41579-021-00630-8, PMID: 34535791 PMC8447893

[ref39] Zhang L. Lin D. Sun X. Curth U. Drosten C. Sauerhering L.. (2020). Crystal structure of SARS-CoV-2 main protease provides a basis for design of improved α-ketoamide inhibitors. Science 368, 409–412. doi: 10.1126/science.abb3405, PMID: 32198291 PMC7164518

[ref40] Zhang R. Yan H. Zhou J. Yan G. Liu X. Shang C.. (2024). Improved fluorescence-based assay for rapid screening and evaluation of SARS-CoV-2 main protease inhibitors. J. Med. Virol. 96:e29498. doi: 10.1002/jmv.29498, PMID: 38436148

[ref41] Zhong N. Zhang S. Xue F. Kang X. Zou P. Chen J.. (2009). C-terminal domain of SARS-CoV main protease can form a 3D domain-swapped dimer. Protein Sci. 18, 839–844. doi: 10.1002/pro.76, PMID: 19319935 PMC2762595

[ref42] Zima V. Radilová K. Kožíšek M. Albiñana C. B. Karlukova E. Brynda J.. (2020). Unraveling the anti-influenza effect of flavonoids: experimental validation of luteolin and its congeners as potent influenza endonuclease inhibitors. Eur. J. Med. Chem. 208:112754. doi: 10.1016/j.ejmech.2020.112754, PMID: 32883638

