# Supplementary material for: Development of a highly sensitive luciferase assay for intracellular evaluation of coronavirus Mpro activity
Source: Front Microbiol. 2025 Apr 2;16:1560251. doi: 10.3389/fmicb.2025.1560251 (PMC12000094; doi:10.3389/fmicb.2025.1560251)
Supplement: Supplementary file 1 [file Data_Sheet_1.docx]

## *Supplementary Material*

# Supplementary Method

## Molecular dynamics simulation

The online tool ProBuilder (<https://www.ddl.unimi.it/vegaol/probuilder.htm>) was utilized to design diverse Mpro substrate peptides corresponding to cleavage sites. Subsequently, molecular docking analyses between the coronavirus Mpro and these synthesized peptides were conducted via the CB-Dock2 software(Liu et al., 2022). Molecular dynamics simulations were conducted using OpenMM, with the initial PDB files repaired using PDBFixer. The Amber14 force field was applied, and the TIP3P (Wang et al., 2014) water model was used, with an ionic strength of 0.15 mol/L to simulate a physiological saline environment. Non-periodic boundary conditions were implemented, with a boundary distance of 1.0 nm. During the simulation, long-range electrostatic interactions were calculated with a 1-nm cutoff for non-bonded interactions. Hydrogen bond constraints were applied to maintain the stability of hydrogen bonds throughout the simulation. The target temperature was set to 310 K, with fluctuations observed during the simulation. The total simulation time was 10 ns, with a timestep of 2 fs. Frames were saved every 5000 steps, for a total of 1000 frames, each containing data on potential energy, kinetic energy, and temperature.

## Supplementary references

Liu, Y., Yang, X., Gan, J., Chen, S., Xiao, Z.-X., and Cao, Y. (2022). CB-Dock2: improved protein–ligand blind docking by integrating cavity detection, docking and homologous template fitting. *Nucleic Acids Res.* 50(W1)**,** W159-W164. doi: 10.1093/nar/gkac394.

Wang, L.P., Martinez, T.J., and Pande, V.S. (2014). Building Force Fields: An Automatic, Systematic, and Reproducible Approach. *J. Phys. Chem. Lett.* 5(11)**,** 1885-1891. doi: 10.1021/jz500737m.

# Supplementary Figures and Tables

## Supplementary Figures
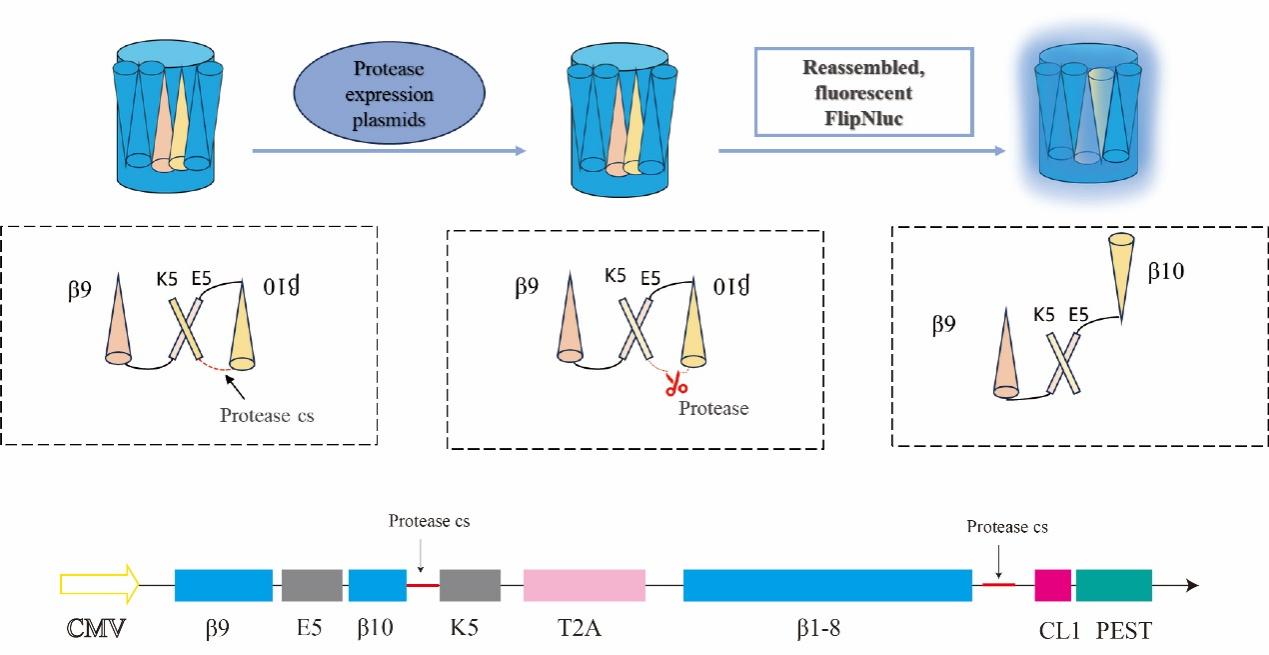


**Supplementary Figure 1**

Schematic structure of the FlipNluc reporter system with a coronavirus Mpro cleavage site. FlipNluc divides Nanoluc into three parts, β1-8, β9, and β10; where β9 and β10 are parallel and consist of heterodimerized coiled-coils E5/K5 and cleavage sequences containing coronavirus cleavage sites. Cleavage of coronavirus Mpro flips its β10 into an antiparallel state with β9, assembling it into a complete Nluc with detectable luciferase activity. At the bottom is the primary structure of the FlipNluc reporter system, which consists of the coronavirus Mpro cleavage sequence followed by a segment of protein degradation sequence (CL1-PEST) connected behind β1-8.

**
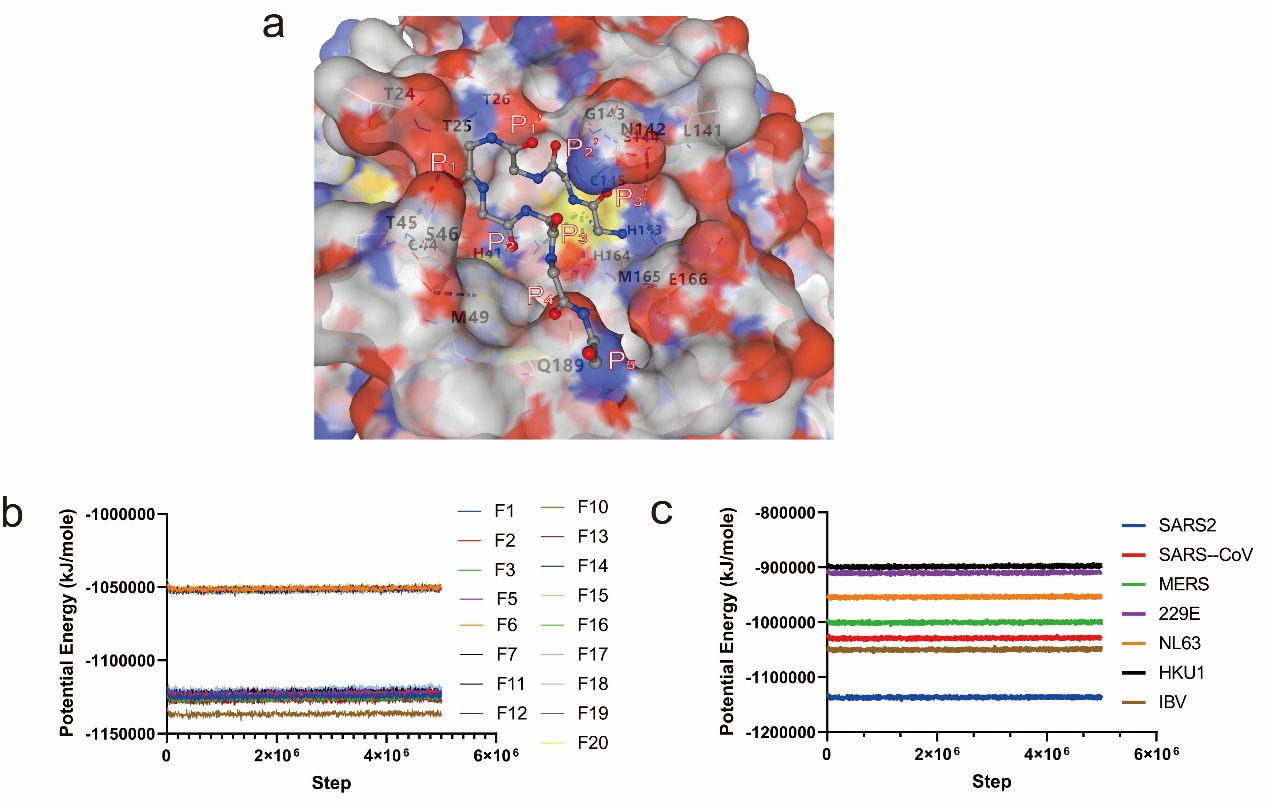
**

**Supplementary Figure 2**

Analysis of coronavirus Mpro with substrate peptides. **a** Crystal structure of SARS-CoV-2 Mpro with a substrate peptide (VAKLQSGF) bound at the active site of both monomers (The peptide was depicted as a grey sticks). **b** MD simulations of SARS-COV-2 Mpro with 17 Mpro substrate peptides (cleavage sites). **c** MD analysis of seven coronavirus Mpro with an Mpro substrate peptide (VAKLQSGF).


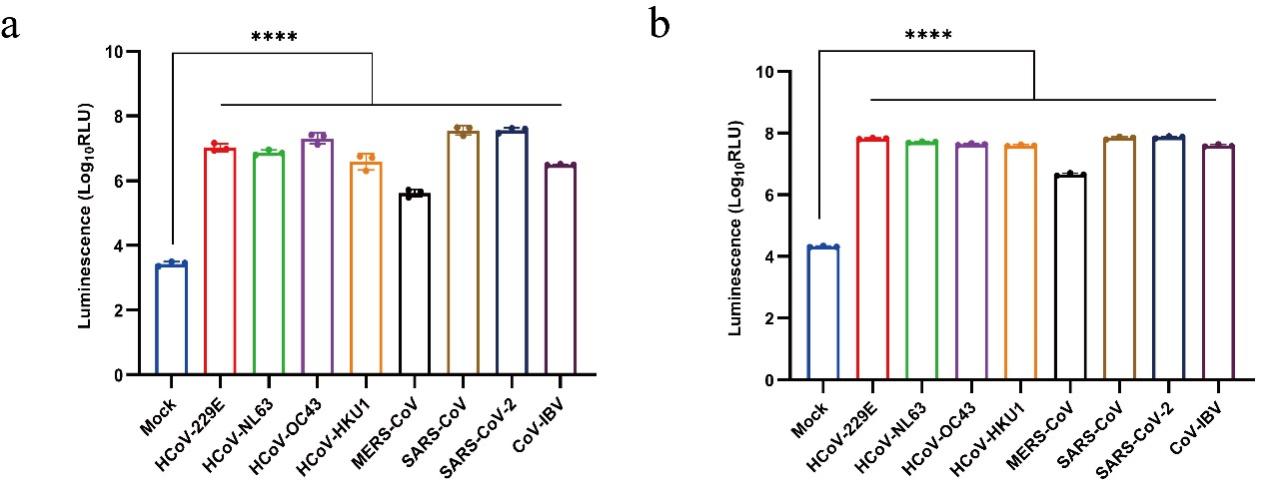


**Supplementary Figure 3**

The ICMP reporter system containing VAVLQSGF cleavage site. ICMP plasmid was co-transfected with eight coronavirus Mpro expression plasmids to determine 24h (**a**) and 48h (**b**) luciferase activity. N=3, Statistically significant differences between experimental groups were determined by analysis of variance (ANOVA) method. ****P*<*0.0001.


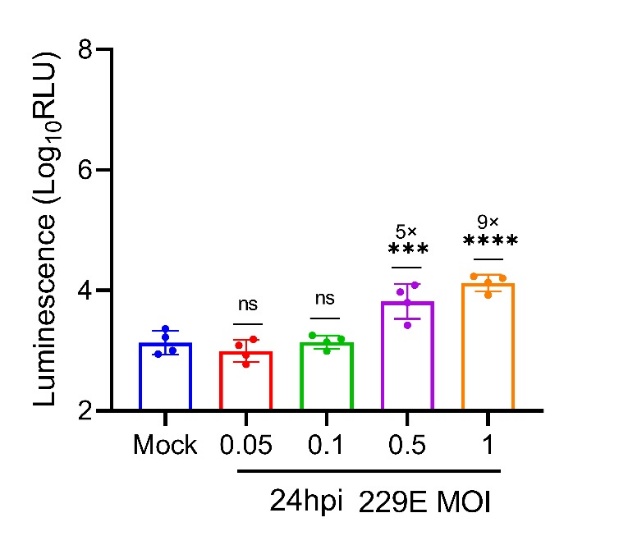


**Supplementary Figure 4**

ICMP transfected Huh7 Cells were infected with HCoV 229E virus at different MOI and fluorescence signals were measured at 24h post-infection. Data were analyzed using one-way ANOVA and error bars represented the mean ± standard error (n=4). ***, P < 0.001, ****, P < 0.0001, ns, P > 0.05).

## Supplementary Tables

## Supplementary Table 1. Design of highly conserved coronavirus Mpro cleavage site sequences

| **Cleavage sites** | **Sequence(P5P4P3P2P1↓P1’P2’P3’)** |
| --- | --- |
| F1 | VARLQSGF |
| F2 | VAVLQSGF |
| F3 | SAVLQSGF |
| F4 | TVRLQSGF |
| F5 | VATLQSGF |
| F6 | TVKLQSGF |
| F7 | TAVLQSGF |
| F8 | VRLQS |
| F9 | AVLQS |
| F10 | VAKLQSGF |
| F11 | SVRLQSGF |
| F12 | VVKLQSGF |
| F13 | VVRLQSGF |
| F14 | FARLQSGF |
| F15 | FAVLQSGF |
| F16 | FAKLQSGF |
| F17 | TAKLQSGF |
| F18 | TARLQSGF |
| F19 | SARLQSGF |
| F20 | SAKLQSGF |

## Supplementary Table 2. Primers used in the construction of plasmid FlipNluc

| Primer | Primer sequence (5ʹ→3ʹ) |
| --- | --- |
| FlipNluc-F | CTTGGTACCGAGCTCGGATCCGCCACCATGGGCTCCCTGCT |
| FlipNluc-R | GGTTTAAACGGGCCCTCTAGATTAGACGTTGATGCGAGCTG |
| FlipNluc2-F | AAATCTCCGTGGCCGTGCTGCAGAGCGGCTTCAAGG |
| FlipNluc2-R | CACGGCCACGGAGATTTTTTTGAAAAGTCGCC |
| FlipNluc3-F | AAATCTCCAGCGCCGTGCTGCAGAGCGGCTTCAAGG |
| FlipNluc3-R | CACGGCGCTGGAGATTTTTTTGAAAAGTCGCC |
| FlipNluc4-F | ATCTCCACGGTGAGACTGCAGAGCGGCTTCAAGG |
| FlipNluc4-R | AGTCTCACCGTGGAGATTTTTTTGAAAAGTCGCC |
| FlipNluc5-F | CACGCTGCAGAGCGGCTTCAAGGTGTCCGCCCTGAAGG |
| FlipNluc5-R | AAGCCGCTCTGCAGCGTGGCCACGGAGATTTTTTTGAAAAGTCGCC |
| FlipNluc6-F | TGAAGCTGCAGAGCGGCTTCAAGGTGTCCGCCCTGAAGG |
| FlipNluc6-R | AGCCGCTCTGCAGCTTCACCGTGGAGATTTTTTTGAAAAGTCGCC |
| FlipNluc7-F | CGTGCTGCAGAGCGGCTTCAAGGTGTCCGCCCTGAAGG |
| FlipNluc7-R | AAGCCGCTCTGCAGCACGGCCGTGGAGATTTTTTTGAAAAGTCGCC |
| FlipNluc8-F | GTGAGACTGCAGAGCAAGGTGTCCGCCCTGAAGG |
| FlipNluc8-R | TTGCTCTGCAGTCTCACGGAGATTTTTTTGAAAAGTCGCC |
| FlipNluc9-F | GCCGTGCTGCAGAGCAAGGTGTCCGCCCTGAAGG |
| FlipNluc9-R | TTGCTCTGCAGCACGGCGGAGATTTTTTTGAAAAGTCGCC |
| FlipNluc10-F | AAATCTCCGTGGCCAAGCTGCAGAGCGGCTTCAAGG |
| FlipNluc10-R | CTTGGCCACGGAGATTTTTTTGAAAAGTCGCC |
| FlipNluc11-F | ATCTCCAGCGTGAGACTGCAGAGCGGCTTCAAGG |
| FlipNluc11-R | AGTCTCACGCTGGAGATTTTTTTGAAAAGTCGCC |
| FlipNluc12-F | ATCTCCGTGGTAAAGCTGCAGAGCGGCTTCAAGG |
| FlipNluc12-R | CAGCTTTACCACGGAGATTTTTTTGAAAAGTCG |
| FlipNluc13-F | ATCTCCGTGGTAAGACTGCAGAGCGGCTTCAAGG |
| FlipNluc13-R | CAGTCTTACCACGGAGATTTTTTTGAAAAGTCG |
| FlipNluc14-F | ATCTCCTTCGCCAGACTGCAGAGCGGCTTCAAGG |
| FlipNluc14-R | AGTCTGGCGAAGGAGATTTTTTTGAAAAGTCGCC |
| FlipNluc15-F | AAATCTCCTTCGCCGTGCTGCAGAGCGGCTTCAAGG |
| FlipNluc15-R | CACGGCGAAGGAGATTTTTTTGAAAAGTCGCC |
| FlipNluc16-F | ATCTCCTTCGCCAAGCTGCAGAGCGGCTTCAAGG |
| FlipNluc16-R | AGCTTGGCGAAGGAGATTTTTTTGAAAAGTCGCC |
| FlipNluc17-F | AAATCTCCACGGCCAAGCTGCAGAGCGGCTTCAAGG |
| FlipNluc17-R | CTTGGCCGTGGAGATTTTTTTGAAAAGTCGCC |
| FlipNluc18-F | AAATCTCCACGGCCAGACTGCAGAGCGGCTTCAAGG |
| FlipNluc18-R | TCTGGCCGTGGAGATTTTTTTGAAAAGTCGCC |
| FlipNluc19-F | AAATCTCCAGCGCCAGACTGCAGAGCGGCTTCAAGG |
| FlipNluc19-R | TCTGGCGCTGGAGATTTTTTTGAAAAGTCGCC |
| FlipNluc20-F | AAATCTCCAGCGCCAAGCTGCAGAGCGGCTTCAAGG |
| FlipNluc20-R | CTTGGCGCTGGAGATTTTTTTGAAAAGTCGCC |

## Supplementary Table 3. Amino acid sequences of synthetic Coronavirus Mpro

| **Coronavirus Mpro** | **GenBank** | **Amino acid sequence** |
| --- | --- | --- |
| HCoV-229E | NC002645 | MAGLRKMAQPSGFVEKCVVRVCYGNTVLNGLWLGDIVYCPRHVIASNTTSAIDYDHEYSIMRLHNFSIISGTAFLGVVGATMHGVTLKIKVSQTNMHTPRHSFRTLKSGEGFNILACYDGCAQGVFGVNMRTNWTIRGSFINGACGSPGYNLKNGEVEFVYMHQIELGSGSHVGSSFDGVMYGGFEDQPNLQVESANQMLTVNVVAFLYAAILNGCTWWLKGEKLFVEHYNEWAQANGFTAMNGEDAFSILAAKTGVCVERLLHAIQVLNNGFGGKQILGYSSLNDEFSINEVVKQMFGVNLQYPYDVPDYA |
| HCoV-NL63 | NC005831 | MSGLKKMAQPSGCVERCVVRVCYGSTVLNGVWLGDTVTCPRHVIAPSTTVLIDYDHAYSTMRLHNFSVSHNGVFLGVVGVTMHGSVLRIKVSQSNVHTPKHVFKTLKPGDSFNILACYEGIASGVFGVNLRTNFTIKGSFINGACGSPGYNVRNDGTVEFCYLHQIELGSGAHVGSDFTGSVYGNFDDQPSLQVESANLMLSDNVVAFLYAALLNGCRWWLCSTRVNVDGFNEWAMANGYTSVSSVECYSILAAKTGVSVEQLLASIQHLHEGFGGKNILGYSSLCDEFTLAEVVKQMYGVNLQYPYDVPDYA |
| HCoV-HKU1 | NC006577 | MSGIVKMVSPTSKIEPCIVSVTYGSMTLNGLWLDDKVYCPRHVICSSSNMNEPDYSALLCRVTLGDFTIMSGRMSLTVVSYQMQGCQLVLTVSLQNPYTPKYTFGNVKPGETFTVLAAYNGRPQGAFHVTMRSSYTIKGSFLCGSCGSVGYVLTGDSVKFVYMHQLELSTGCHTGTDFTGNFYGPYRDAQVVQLPVKDYVQTVNVIAWLYAAILNNCAWFVQNDVCSTEDFNVWAMANGFSQVKADLVLDALASMTGVSIETLLAAIKRLYMGFQGRQILGSCTFEDELAPSDVYQQLAGVKLQYPYDVPDYA |
| HCoV-OC43 | NC006213 | MSGIVKMVNPTSKVEPCVVSVTYGNMTLNGLWLDDKVYCPRHVICSASDMTNPDYTNLLCRVTSSDFTVLFDRLSLTVMSYQMRGCMLVLTVTLQNSRTPKYTFGVVKPGETFTVLAAYNGKPQGAFHVTMRSSYTIKGSFLCGSCGSVGYVIMGDCVKFVYMHQLELSTGCHTGTDFNGDFYGPYKDAQVVQLLIQDYIQSVNFVAWLYAAILNNCNWFVQSDKCSVEDFNVWALSNGFSQVKSDLVIDALASMTGVSLETLLAAIKRLKNGFQGRQIMGSCSFEDELTPSDVYQQLAGIKLQDIYPYDVPDYA |
| MERS-CoV | NC019843 | MSGLVKMSHPSGDVEACMVQVTCGSMTLNGLWLDNTVWCPRHVMCPADQLSDPNYDALLISMTNHSFSVQKHIGAPANLRVVGHAMQGTLLKLTVDVANPSTPAYTFTTVKPGAAFSVLACYNGRPTGTFTVVMRPNYTIKGSFLCGSCGSVGYTKEGSVINFCYMHQMELANGTHTGSAFDGTMYGAFMDKQVHQVQLTDKYCSVNVVAWLYAAILNGCAWFVKPNRTSVVSFNEWALANQFTEFVGTQSVDMLAVKTGVAIEQLLYAIQQLYTGFQGKQILGSTMLEDEFTPEDVNMQIMGVVMQYPYDVPDYA |
| SARS-CoV | NC004718 | MSGFRKMAFPSGKVEGCMVQVTCGTTTLNGLWLDDTVYCPRHVICTAEDMLNPNYEDLLIRKSNHSFLVQAGNVQLRVIGHSMQNCLLRLKVDTSNPKTPKYKFVRIQPGQTFSVLACYNGSPSGVYQCAMRPNHTIKGSFLNGSCGSVGFNIDYDCVSFCYMHHMELPTGVHAGTDLEGKFYGPFVDRQTAQAAGTDTTITLNVLAWLYAAVINGDRWFLNRFTTTLNDFNLVAMKYNYEPLTQDHVDILGPLSAQTGIAVLDMCAALKELLQNGMNGRTILGSTILEDEFTPFDVVRQCSGVTFQDIYPYDVPDYA |
| SARS-CoV-2 | NC045512 | MSGFRKMAFPSGKVEGCMVQVTCGTTTLNGLWLDDVVYCPRHVICTSEDMLNPNYEDLLIRKSNHNFLVQAGNVQLRVIGHSMQNCVLKLKVDTANPKTPKYKFVRIQPGQTFSVLACYNGSPSGVYQCAMRPNFTIKGSFLNGSCGSVGFNIDYDCVSFCYMHHMELPTGVHAGTDLEGNFYGPFVDRQTAQAAGTDTTITVNVLAWLYAAVINGDRWFLNRFTTTLNDFNLVAMKYNYEPLTQDHVDILGPLSAQTGIAVLDMCASLKELLQNGMNGRTILGSALLEDEFTPFDVVRQCSGVTFQYPYDVPDYA |
| CoV-IBV | NC001451 | MSGFKKLVSPSSAVEKCIVSVSYRGNNLNGLWLGDTIYCPRHVLGKFSGDQWNDVLNLANNHEFEVTTQHGVTLNVVSRRLKGAVLILQTAVANAETPKYKFIKANCGDSFTIACAYGGTVVGLYPVTMRSNGTIRASFLAGACGSVGFNIEKGVVNFFYMHHLELPNALHTGTDLMGEFYGGYVDEEVAQRVPPDNLVTNNIVAWLYAAIISVKESSFSLPKWLESTTVSVDDYNKWAGDNGFTPFSTSTAITKLSAITGVDVCKLLRTIMVKNSQWGGDPILGQYNFEDELTPESVFNQIGGVRLQYPYDVPDYA |
| MHV | NC048217 | MSGIVKMVSPTSKVEPCIVSVTYGNMTLNGLWLDDKVYCPRHVICSSADMTDPDYPNLLCRVTSSDFCVMSGRMSLTVMSYQMQGCQLVLTVTLQNPNTPKYSFGVVKPGETFTVLAAYNGRPQGAFHVTLRSSHTIKGSFLCGSCGSVGYVLTGDSVRFVYMHQLELSTGCHTGTDFSGNFYGPYRDAQVVQLPVQDYTQTVNVVAWLYAAIFNRCNWFVQSDSCSLEEFNVWAMTNGFSSIKADLVLDALASMTGVTVEQVLAAIKRLHSGFQGKQILGSCVLEDELTPSDVYQQLAGVKLQYPYDVPDYA |
| PEDV | NC003436 | MAGLRKMAQPSGVVEKCIVRVCYGNMALNGLWLGDIVMCPRHVIASSTTSTIDYDYALSVLRLHNFSISSGNVFLGVVSATMRGALLQIKVNQNNVHTPKYTYRTVRPGESFNILACYDGAAAGVYGVNMRSNYTIRGSFINGACGSPGYNINNGTVEFCYLHQLELGSGCHVGSDLDGVMYGGYEDQPTLQVEGASSLFTENVLAFLYAALINGSTWWLSSSRIAVDRFNEWAVHNGMTTVGNTDCFSILAAKTGVDVQRLLASIQSLHKNFGGKQILGHTSLTDEFTTGEVVRQMYGVNLQYPYDVPDYA |
| PDCoV | KJ601778 | MAGIKILLHPSGVVERCMVSVVYNGSALNGIWLKNVVYCPRHVIGKFRGDQWTHMVSIADCRDFIVKCPIQGIQLNVQSVKMVGALLQLTVHTNNTATPDYKFERLQPGSSMTIACAYDGIVRHVYHVVLQLNNLIYASFLNGACGSVGYTLKGKTLYLHYMHHIEFNNKTHSGTDLEGNFYGPYVDEEVIQQQTAFQYYTDNVVAQLYAHLLTVDARPKWLAQSQISIEDFNSWAANNSFANFPCEQTNMSYIMGLSQTARVPVERILNTIIQLTTNRDGACIMGSYDFECDWTPEMVYNQAPISLQSYPYDVPDYA |
| HKU19 | NC016994 | MAGIKRLLSPSGSVEKCMVSVHYRGLTLNGIWLNNVIYCPRHILGKYQASFWQDAVKVADTRDFVINSQHSKIQFRPVGLRLNNAILQIVLPTEQNNPHTPDYEFVTAKPGSSMTIACTYDGIVSAIYHVIMQTNGLIYASFMNGACGSVGYTLKNGKLLLHYMHHLEFNNKTHGGTDLNGNFYGDYIDEEIAQSISKAATLTDNALAHIYAHLSTISTKPKWLSYQELSVEDFNDWAKNNDHTQFPSCDENYTYLDALAKSTGVSIKRVLSTLVTLHTNWGSASVLGMSTFDLDFTPEMVYNQAPITLQGYPYDVPDYA |
| HKU14 | NC017083 | MSGIVKMVSPTSKVEPCVVSVTYGNMTLNGLWLDDKVYCPRHVICSASDMTNPDYPNLLCRVTSSDFTIMSDRMSLTVMSYQMQGCMLVLTVTLQNPRTPKYTFGVVKPGETFTVLAAYNGRPQGAFHVTMRSSFTIKGSFLCGSCGSVGYVLMGDCVKFVYMHQLELSTGCHTGTDFNGDFYGPYKDAQVVQLPVQDYVQSVNFVAWLYAAILNNCNWFVQSDRCSIEDYNVWAMSNGFSQIKSDLVVDALASMTGVSLENLLAAIKRLHKGFQGRQIMGSCAFEDELTPSDVYQQLAGVKLQYPYDVPDYA |
| SARS-CoV-2 C145A mutant | This work | MSGFRKMAFPSGKVEGCMVQVTCGTTTLNGLWLDDVVYCPRHVICTSEDMLNPNYEDLLIRKSNHNFLVQAGNVQLRVIGHSMQNCVLKLKVDTANPKTPKYKFVRIQPGQTFSVLACYNGSPSGVYQCAMRPNFTIKGSFLNGSAGSVGFNIDYDCVSFCYMHHMELPTGVHAGTDLEGNFYGPFVDRQTAQAAGTDTTITVNVLAWLYAAVINGDRWFLNRFTTTLNDFNLVAMKYNYEPLTQDHVDILGPLSAQTGIAVLDMCASLKELLQNGMNGRTILGSALLEDEFTPFDVVRQCSGVTFQYPYDVPDYA |

Note: Amino Acids in red indicates HA label

## Supplementary Table 4. ICMP reporter gene sequence

| **Reporter Gene** | **Amino acid sequence** |
| --- | --- |
| ICMP | ATMVSGWELFKKISGGGGSVAKLQSGFSGGGGSVTGYRLFEEILSSGSGGGGSGGGGSGGGGSGGGGSGGGGSGGGGSGGGGSGGGGSGGGGSVFTLEDFVGDWEQTAAYNLDQVLEQGGVSSLLQNLAVSVTPIQRIVRSGENALKIDIHVIIPYEGLSADQMAQIEEVFKVVYPVDDHHFKVILPYGTLVIDGVTPNMLNYFGRPYEGIAVFDGKKITVTGTLWNGNKIIDERLITPDGSMLFRVTINSGGGGSGGGGSVAKLQSGFSGGGGSVSGWDLFKKIS |

Note: Amino Acids in red indicates Mpro cleavage site
